# Safety and Engraftment of Aligned Cardiac Patches Loaded with hiPSC-CMs in a Large Animal Model of Myocardial Infarction

**DOI:** 10.7150/thno.121105

**Published:** 2026-01-14

**Authors:** Divya Sridharan, Salman Pervaiz, Nikita C. Nair, Muhamad M. Mergaye, Helena Islam, Britani N Blackstone, Syed A. Ashraf, Syed B. Alvi, Matthew Joseph, Juliet Varghese, Yuchi Han, Orlando P. Simonetti, Heather M. Powell, Konstantinos Dean Boudoulas, Robert L. Hamlin, Mahmood Khan

**Affiliations:** 1 Division of Basic and Translational Sciences, Department of Emergency Medicine, The Ohio State University, Columbus, OH, United States.; 2 Dorothy M. Davis Heart and Lung Research Institute, The Ohio State University, Columbus, OH, United States.; 3 Division of Cardiovascular Medicine, Department of Internal Medicine, The Ohio State University, Columbus, OH, United States.; 4 Department of Biomedical Engineering, The Ohio State University, Columbus, OH, United States.; 5 Department of Radiology, The Ohio State University, Columbus, OH, United States.; 6 Department of Veterinary Sciences, The Ohio State University, Columbus, OH, United States.

**Keywords:** preclinical, porcine, cardiac patch, stem cell transplantation, myocardial infarction, induced pluripotent stem cell-derived cardiomyocytes

## Abstract

Myocardial infarction (MI) is a leading cause of death in the United States. Human induced pluripotent stem cell-derived cardiomyocytes (hiPSC-CMs) present a promising strategy for regenerating the damaged heart tissue post-MI. However, poor cell engraftment and survival remain significant barriers in their effective use for myocardial repair. In this study, we developed a “cardiac patch” using a bi-layered, aligned coaxial patch for epicardial delivery of hiPSC-CMs in a preclinical porcine MI model. The cardiac patch (40 mm in diameter and 500 µm thick) was fabricated using polycaprolactone (PCL) and gelatin via electrospinning and seeded with twenty-two million hiPSC-CMs. In vitro functional assessment showed synchronized contractility of the hiPSC-CMs along the aligned fibers. The in vivo transplantation of the cardiac patch was performed in a translationally relevant preclinical large animal (porcine) MI model at 1-week after MI induction. Histological assessments showed successful engraftment and survival of the hiPSC-CMs at the infarct, up to 4-weeks after cardiac patch-transplantation. This was accompanied by modest improvements in LVEF (Patch:18.0% vs Control: -1.2%) and a decrease in the enhancement percentage (Patch: 28.8% vs Control: 18.6%) at 4-weeks post-patch transplantation. Additionally, absence of arrhythmias or teratoma formation, affirmed the safety of the cardiac patch. Overall, we have demonstrated the feasibility, safety and engraftment of bi-layered aligned cardiac patches seeded with hiPSC-CMs in preclinical porcine MI model as a promising therapeutic approach for myocardial regeneration post-MI.

## 1. Introduction

Acute myocardial infarction (MI) leading to heart failure is a major contributor to patient morbidity and mortality in the United States^1^. MI occurs because of blockage in the epicardial coronary vasculature leading to partial or complete blockage in the blood flow to the left ventricle. Consequently, the lack of oxygen and nutrients induces an ischemic microenvironment in the myocardium leading to significant cardiomyocyte apoptosis^2^. The extensive myocardial remodeling observed after MI involves the replacement of necrotic myocardium by fibrotic scar tissue^3^, ^4^. This collagen-rich scar tissue is non-contractile and therefore compromises the cardiac function resulting in heart failure. Current therapies for the management of MI, like percutaneous coronary intervention (PCI), fibrinolysis, and coronary bypass grafting (CABG), have been successful in decreasing the morbidity and mortality in patients^5^. However, these modalities are palliative and fail to repair and regenerate the damaged myocardium. Hence, the major focus of cardiovascular regenerative medicine over the last two decades has been to develop therapeutic strategies to regenerate the myocardium after MI.

The advent of stem cells, especially human induced pluripotent stem cells (hiPSCs) was a critical landmark in the field of cell therapy and regenerative medicine^4^. Transplantation of hiPSC-derived cardiomyocytes (hiPSC-CMs) has been shown to improve the function of damaged myocardium after MI in several preclinical studies^4^, ^6^. Transplanted hiPSC-CMs have been shown to promote angiogenesis, enhance cardioprotection, and reduce myocardial remodeling in the ischemic myocardium^7^. However, the potential risk of teratoma formation associated with the use of cells differentiated from hiPSCs, the poor engraftment, and the long-term survival of the transplanted cells in the ischemic myocardium have been the major roadblocks in the successful translation of hiPSC-CM-based cell therapies from bench to bedside^8^, ^9^.

In the last decade, engineered heart tissues (EHTs) have been developed using a combination of bioengineered scaffolds and hiPSC-derived differentiated cells^10^. These EHTs have been shown to improve the engraftment and long-term survival of hiPSC-CMs in preclinical animal studies. While significant progress has been made in the use of EHTs for cell transplantation, there is still a need to develop the “ideal” scaffold to mimic the structure, cellular composition, and mechanical properties of the myocardium^11^. Different biomaterials including natural polymers like gelatin, collagen and fibrin-thrombin, as well as synthetic polymers like polycaprolactone (PCL), polydimethylsiloxane (PDMS), and poly(L-lactic acid) (PLA) and poly (lactic-co-glycolic acid) (PLGA) have been used to fabricate scaffolds for cardiac cell transplantation due to their superior biocompatibility and mechanical properties, respectively^12^.

Recently, our group fabricated a biomimetic aligned coaxial PCL-gelatin scaffold with a PCL-core and gelatin-shell^11^. This unique “core-shell” structure enables the patch to harness the high biocompatibility of gelatin without compromising on the mechanical strength of PCL. Moreover, aligned fibers resulted in the parallelly-arranged, rod-shaped hiPSC-CMs, which closely resembled the morphology and arrangement of cardiomyocytes of the adult myocardium. While the use of coaxial PCL-gelatin electrospun patches has been used for wound healing^13^, vascular^14^ and bone^15^ tissue engineering applications, the use of these scaffolds for cardiac regenerative applications is yet to be explored. In the present study, we developed a large bi-layered cardiac patch by culturing hiPSC-CMs on these aligned coaxial PCL-gelatin scaffolds. We assessed the survival, retention, and engraftment of the hiPSC-CMs transplanted using this cardiac patch in a preclinical large animal porcine model of MI. Furthermore, we assessed the instances of arrhythmia and teratoma formation in these pigs to evaluate the safety of these cardiac patches.

## 2. Materials and Methods

### 2.1. Fabrication of PCL-gelatin patch

Aligned PCL-gelatin patches were fabricated as previously described^16^, ^17^. Briefly, gelatin (12% w/v) (Sigma-Aldrich) and PCL (8% w/v) (Sigma-Aldrich) solutions were prepared in 1,1,1,3,3,3-hexafluoro-2-propanol. The PCL and gelatin solutions were fed to the inner (1 ml/h) and outer tube (4 ml/h), respectively, of the coaxial spinneret. The aligned coaxial fibers were collected onto a grounded rotating collector and dried inside a chemical fume hood overnight. The thickness of the patches was regulated by electrospinning for a predetermined time duration. The patch thickness was verified to be within ± 20 µm of the desired thickness (either 200 or 500 µm) using digital calipers (Fisher Scientific) to measure three 8 mm biopsy punches of the scaffold taken from the center and the two edges of the scaffold. The patches were crosslinked using 7 mM 1-ethyl-3-(3Dimethylamino propyl)carbodiimide hydrochloride (EDC) solution in ethanol for 24 h, followed by sterilization with 70% ethanol, and hydration in PBS^16^, ^17^.

### 2.2. Attenuated total reflectance-fourier transform infrared spectroscopy (ATR-FTIR)

To validate the coaxial structure of the scaffolds, pure gelatin, pure PCL, gelatin-PCL blend, and PCL-gelatin core-shell scaffolds were analyzed with ATR-FTIR via a Nicolet Summit with the Everest ATR attachment and a zinc-selenide crystal. Each scaffold type was scanned 50 times at a resolution of 8 cm^-1^, then averaged by the equipped OMNIC Paradigm software. Absorbance peaks of scaffolds were assessed qualitatively by comparing expected peaks for electrospun PCL (1724cm^-1^, 1240 cm^-1^, and 1190 cm^-1^) and gelatin (1650cm^-1^ and 1540cm^-1^) to blend and coaxial scaffolds^18^. Representative wavenumber (cm^-1^) vs. absorbance plots were selected for each scaffold type, and data shown only from 3800 cm^-1^ to 600 cm^-1^ to enhance peak detail for qualitative comparison.

### 2.3. Mechanical testing of PCL-gelatin cardiac patch

The mechanical properties of the two scaffold thicknesses were assessed via tensile testing. Samples were standardized with a dog bone-shaped punch (4 mm width, 21 mm gauge length). Two punches were stacked for each sample, thickness was measured via digital calipers, and the samples were strained until failure at a rate of 2 mm/s (TestResources 100R, Shakopee, MN, USA) in the longitudinal or transverse direction of fiber alignment (n=6 for each condition). Maximum load at failure and linear stiffness, calculated via linear regression analysis of the first linear region (R^2^ ≥ 0.95), were determined from load-position curves. Ultimate tensile strength, elastic modulus, and toughness were calculated from stress-strain curves as described previously^19^.

### 2.4. Cell culture

The hiPSC-CMs were procured from Fujifilm Cellular Dynamics International. The cells were plated in 0.1% gelatin-coated six-well plates as previously described per the manufacturer's protocol. After one-week, the hiPSC-CMs were seeded onto aligned coaxial patches as previously described^16^, ^17^. Briefly, the PCL-gelatin patches were cut into circular patches of diameter 8 mm and 40 mm for *in vitro* and in vivo studies, respectively. The hiPSC-CMs were trypsinized, counted, and seeded onto the first side of the patches at a final density of 1 × 10^6^ cells/cm^2^ and incubated overnight at 5% CO_2_ at 37°C. The following day, the patches were flipped over, and hiPSC-CMs were seeded on the second side of the patch as described above. The hiPSC-CM-seeded cardiac patches were cultured in cardiomyocyte maintenance medium as described previously^16^.

### 2.5. Animal husbandry

Seven immunocompetent male Yorkshire pigs weighing 25-30 kg having a mean age of 2-3 months (**Supplementary Table 1**) at the time of MI induction, were used in our experimental study. Two pigs died shortly after the MI induction procedure due to fatal arrhythmias. Of the remaining five pigs, three underwent transplantation of patches seeded with hiPSC-CMs (MI + patch), and two underwent MI induction without receiving a patch (MI only). Animals were inspected upon arrival at the housing facility and during the time of the experiment according to the Ohio State University Laboratory and Animal Resources (OSU-ULAR) standard protocols.

### 2.7. Induction of myocardial infarction in pigs

MI induction surgery was performed using standard sterile techniques in accordance with OSU Institutional Animal Care and Use Committee (IACUC) guidelines. The surgery was performed in a sterile suite. (**Supplementary Fig. 1A-C**) Upon induction of anesthesia with an intramuscular injection of ketamine (16.5 mg/kg) and acepromazine (0.6 mg/kg) the pigs were intubated, placed on a ventilator, and maintained with 2-3% isoflurane. The adequacy of anesthesia was confirmed by loss of reflexes, muscle response, loss of response to stimuli while under anesthesia. Bupivacaine (0.5%) was administered at the site of the incision. Animals were positioned supine, an incision was made in the groin, the right femoral artery exposed, and 7F (7 French) arterial sheath was introduced for arterial access. The animal was anticoagulated with heparin (100 IU/kg) and amiodarone (100 mg bolus); was administered as an antiarrhythmic. EKG and blood pressure were closely monitored during the entire experimental protocol. Under fluoroscopic guidance a guiding catheter (7F AL 0.75 STSH) was advanced up the descending aorta, around the aortic arch and into the left main coronary ostium. A baseline coronary angiogram was recorded to identify the location of diagonal branches and to select an occlusion site. Next, a 0.014” guide wire was advanced through the guide catheter and into the distal LAD. A 3.5 mm x 8 mm coronary angioplasty balloon was advanced over the wire, positioned distal to the first diagonal branch, and inflated to occlude blood flow to the distal LAD. Complete occlusion was confirmed by coronary angiogram. After 90 minutes of ischemia the balloon was deflated, removed from the vessel, and restoration of flow confirmed with an angiogram. At the conclusion of the procedure, the sheath was removed, the femoral vessel was ligated, and the muscle layers, subcutaneous tissue, and skin were closed in layers using 3-0 Vicryl.

### 2.8. Epicardial cardiac patch transplantation post-MI

One-week after the MI induction, by means of a left thoracotomy, the heart was exposed, the infarcted region was grossly identified, and the cell-seeded bi-layered cardiac patches was secured to the epicardial heart surface on the periphery of the infarct region using a 2-0 silk suture, at four points (**Supplementary Fig. 1D-I**). To prevent transplant rejection, an immunosuppression regime was initiated with tacrolimus capsules (1 mg/kg) administered once daily, starting on the day of MI surgery and continuing until the study's conclusion. On the day of sham/ patch-transplantation surgery, a 5 mg bolus of tacrolimus was given prior to the surgery. Immediately after the sham/transplantation surgery, a single 500 mg bolus of methylprednisolone was administered in each pig. Additionally, oral methylprednisolone (1 mg/kg) was prescribed for two weeks, beginning on the first post-operative day. The immunosuppression regime was given to both the control and patch-transplanted pigs.

### 2.9. Electrocardiogram analysis

The electrocardiogram (ECG) was recorded in the pigs to assess the changes in electrical activity of the heart during and after cardiac patch transplantation. ECG was recorded using the AD Instruments PowerLab 4/20. The ECG signals were recorded continuously using the Labchart software 8. The ECG was recorded at baseline, during ischemia, reperfusion, 1-week post-MI and at 4-weeks after cardiac patch transplantation as previously described^20^. Each ECG was analyzed using the Labchart 8 software to determine the presence of cardiac arrhythmias at different time points in the study duration.

### 2.10. Cardiac magnetic resonance imaging

To assess the functional impact of cardiac patch and determine the presence and extent of infarcted myocardium, the animals (MI only; n=2 and MI + patch; n=3) were imaged at two time points: 1-week post-MI (just before transplanting the patch) and 5-weeks post-MI on a 0.55T MR system (MAGNETOM FreeMax, Siemens Healthineers, Forchheim, Germany)^21^-^23^. A breath-held segmented balanced steady state free precession (bSSFP) sequence with compressed sensing acceleration was used to acquire cine images with a temporal resolution of 19ms. A stack of cine short-axis slices covering the left ventricle was acquired to assess left ventricular function. An ECG-triggered, breath-held, inversion recovery prepared segmented bSSFP research sequence was used for late gadolinium enhanced (LGE) imaging to visualize the infarct. A stack of 2D LGE short-axis images covering the ventricle was acquired post-injection of 0.15 - 0.20 mmol/kg of the gadolinium-based MRI contrast agent gadobutrol (Gadavist, Bayer Healthcare, Whippany, NJ, USA). Additionally, cine and LGE images were also acquired along long-axis views (two, three and four chamber) to provide visual guidance in the quantitative analysis. The MR image analysis to determine the cardiac function from the cine images and the extent of infarct from the LGE images was performed using the suiteHEART software (NeoSoft LLC, Pewaukee, WI). Specifically, endocardial and epicardial contours were automatically generated for cine and LGE short-axis images, respectively and manually adjusted as needed for accurate measurement. For LGE images, the full-width-half maximum method was used to identify the infarcted myocardium relative to the healthy or remote myocardium. Cardiac volumes were normalized to body surface area to account for the rapid weight gain of pigs between imaging time points. BSA was estimated using the Meeh's formula^24^.

### 2.11. Transmission electron microscopy (TEM)

TEM imaging was performed on the cross-linked fibers after subjecting them to mild disintegration using a probe sonicator (Fisher Scientific, Sonic dismembrator 500), and later drop-casting the samples on a copper grid for imaging using the FEI Tecnai G2 Biotwin TEM at 80 kV, and micrographs were captured using an AMT camera.

### 2.12. Scanning electron microscopy (SEM)

To assess the morphology, the cross-linked aligned fiber patches were cut into 8 mm circular patches and were mounted onto SEM stubs using carbon tape and sputter-coated with gold-palladium (Pelco Model 3). The patches were then imaged on the Apreo SEM (Thermo Scientific) at 10 kV. The images acquired were analyzed using ImageJ software (Version 1.53) to measure the fiber diameter, pore size, and pore area (6 FOV and 60 fibers per FOV, per condition), as previously described^25^.

### 2.13. Immunofluorescence staining

For whole-mount immunostaining, the hiPSC-CMs were cultured on the aligned fiber patches and processed as previously described^11^. Briefly, the patches were fixed using 4% paraformaldehyde, permeabilized using 0.2% Triton X-100, and incubated in 1% bovine serum albumin (BSA, in PBS) to block non-specific binding. The patches were then incubated with the primary antibody overnight at 4 °C followed by the corresponding secondary antibodies for 1h at room temperature. Finally, the patches were counterstained with DAPI and imaged on the Olympus FV3000 microscope.

For immunohistochemical staining of cardiac patch sections and porcine heart sections, the samples were fixed in 4% PFA overnight at 4 °C. The samples were then incubated in 10% sucrose and 30% sucrose for 24h each to dehydrate the samples before embedding them in OCT compound. The OCT blocks were then cut into 8 micron-thick sections on the cryotome and mounted onto Gold Plus glass slides. The sections were then washed to remove excess OCT and permeabilized using 0.2% Triton X-100 and non-specific antibody binding was blocked using 10% normal goat serum (NGS, in PBS). The sections were then incubated in primary antibodies overnight at 4 °C followed by the corresponding secondary antibodies. The sections were counterstained with DAPI, and coverslips were mounted using the Prolong Gold Antifade. All incubations were followed by three washes with PBS. The sections were imaged using the Olympus FV3000 microscope.

The antibodies used in the study are: anti-cardiac Troponin T antibody (Sigma, Cat No. HPA017888, 1:1000), anti-human nuclei antibody-Cy3 (Sigma, Cat No. MAB1281C3, 1:100) anti-GATA4 antibody (Thermofisher, Cat No. PA1-102, 1:500), anti-α-sarcomeric actinin (Sigma, Cat No. A7732, 1:500), Goat anti-mouse IgG Alexa Fluor 488 (Thermofisher, Cat No. A11001, 1:1000), Goat anti-rabbit IgG Alexa Flour 488 (Thermofisher, Cat No. A11008, 1:1000), Goat anti-rabbit IgG Alexa Fluor 594 (Thermofisher, Cat No. A11012, 1:2000).

### 2.14. Calcium imaging

The calcium transients in the hiPSC-CMs cultured on the patches were assessed to determine the functionality of the cells as previously described^26^. Briefly, the patches were stained with 10 µM of Fluo4-AM and imaged using the line scan mode on the Nikon A1R microscope. The line scan images were then quantified using ImageJ as previously described^26^.

### 2.15. Multielectrode array

The electrophysiological properties of the cardiac patches were assessed using MEA analysis as previously described^11^. Briefly, the cell-seeded cardiac patches were cultured for four days and the field potentials of the hiPSC-CMs were recorded using the Axion Biosystems MEA system. The response of the hiPSC-CMs to isoproterenol (ISO) was assessed by incubating the patches in medium supplemented with 10 nM and 100 nM of ISO as previously described^11^.

### 2.16. Masson trichrome staining

The extent of fibrosis in the cardiac tissue sections was assessed by performing Masson-Trichrome staining as previously described^27^. Briefly, the tissues were embedded in OCT and sectioned using the Leica CM1950 cryostat. The cryosections were then stained using the Masson Trichrome Staining kit (Sigma-Aldrich) and imaged on a brightfield microscope.

### 2.17. Statistical analysis

All values are presented as mean values ± standard error. The statistical significance between the groups was determined by the student's t-test. Values were considered significant if the P values were < 0.05. All statistical analyses were performed using GraphPad Prism Software, version 10.

## 3. Results

### 3.1. *In vitro* characterization of aligned fiber patch

**Figure 1:**
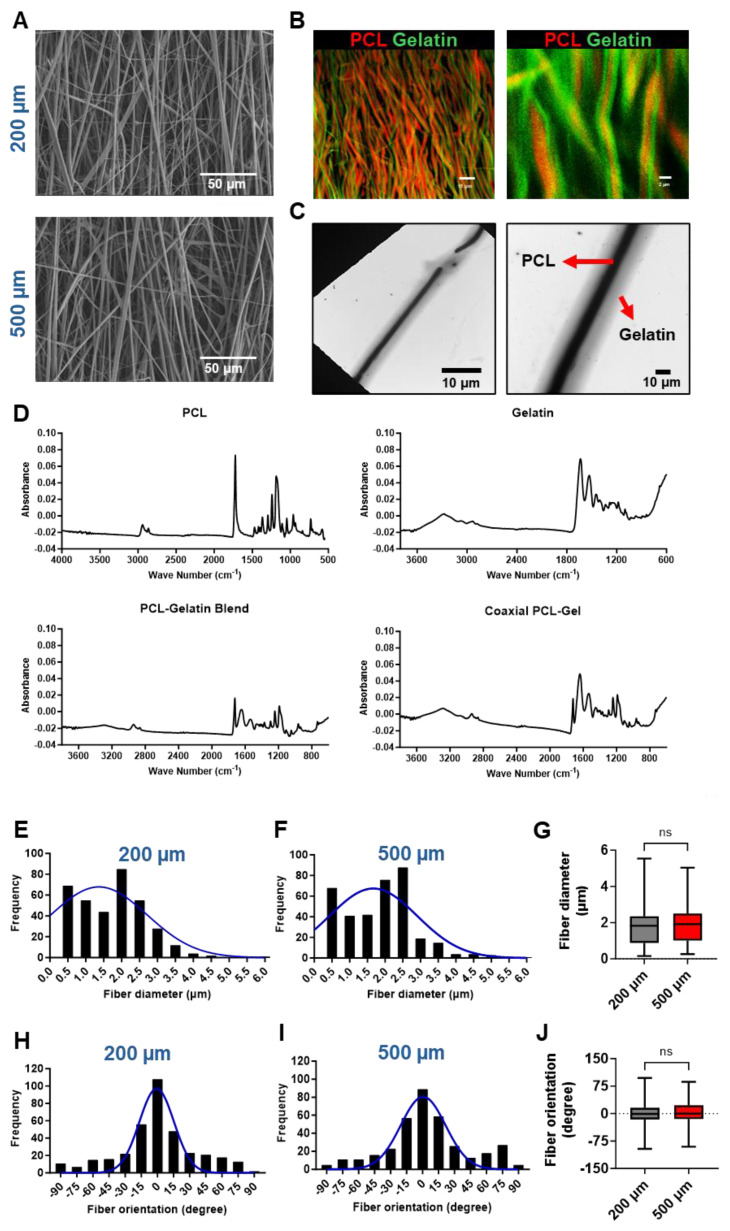
**Characterization of PCL-gelatin coaxial patch.** Representative SEM images of **(A)** 200 µm and 500 µm thick coaxial patches. Scale: 10 µm. **(B)** Fluorescence images showing the coaxial structure of fibers with PCL core (red) and gelatin shell (green). Scale: 15 µm, 2 µm. **(C)** TEM images showing the core-shell structure of each fiber. Scale: 10 µm. **(D)** ATR-FTIR data showing the characterization of PCL, gelatin, PCL-gelatin blend, and PCL-gelatin core-shell fibers. **(E-F)** Frequency distribution and **(G)** mean of nanofiber diameter in 200 µm and 500 µm thick patches. **(H-I)** Frequency distribution and **(J)** mean of fiber orientation in 200 µm and 500 µm patches. **(E-J)** N= 360 fibers from 6 different patches.

Aligned fiber PCL-gelatin coaxial patches of two different thicknesses, 200 µm and 500 µm, were fabricated via electrospinning (**Fig. 1**). The fibers had a coaxial morphology with a PCL core (stained with rhodamine) and a gelatin shell (stained with fluorescein, **Fig. 1B**). Moreover, TEM images confirmed the coaxial structure of the fibers, with a clear distinction between the PCL core and gelatin shell (**Fig. 1C**).

To confirm the separation in the biomaterials in the core and shell, ATR-FTIR spectroscopy was performed (**Fig. 1D**). ATR-FTIR of PCL yielded strong peaks at 1722cm^-1^ (C = O stretch), 1241cm^-1^, and 1185cm^-1^ with weaker peaks at 2941cm^-1^, 2864cm^-1^ (C-H stretch), and 960cm^-1^ (C-O-C or C-C stretch). Additionally, gelatin was observed by strong peaks at 1635cm^-1^ and 1529cm^-1^ (N-H), and additional broad peaks at 3274cm^-1^ (N-H and O-H stretch) and 2934cm^-1^ (C-H stretch). Generally, blend and coaxial scaffolds had reduced absorbance for all peaks compared to the pure samples, but the background signal was still minimal. Blend scaffolds contained strong, expected peaks for PCL (1723cm^-1^, 1242cm^-1^, and 1190cm^-1^) and gelatin (1641cm^-1^ and 1543cm^-1^) with additional weak (2939cm^-1^, 2865cm^-1^, and 961cm^-1^) and broad (3296cm^-1^) peaks. Similarly, coaxial scaffolds contained strong PCL (1722cm^-1^, 1242cm^-1^, and 1189cm^-1^) and gelatin (1639cm^-1^ and 1534cm^-1^) peaks with observed weak (2937cm^-1^ and 961cm^-1^) and broad (3288cm^-1^) peaks. It is worth noting that in the blend, the PCL carbonyl peak was stronger than the Gelatin carbonyl peak. Conversely, in the coaxial gel, the Gelatin carbonyl peak was stronger than the PCL carbonyl peak.

The fibers in the 200 µm and 500 µm thick scaffolds had a mean fiber diameter of 1.732 ± 0.05 µm and 1.839 ± 0.05 µm, respectively (**Fig. 1E-G**). We did not observe any significant differences in the distribution or mean fiber diameter between the two scaffolds. Similarly, most of the fibers in the 200 and 500 µm scaffolds were arranged parallel to one another, with no significant differences in the distribution or mean fiber orientations (**Fig. 1H-J**). Taken together, our observations showed no significant differences in the morphology of the scaffolds irrespective of their overall thickness.

**Figure 2:**
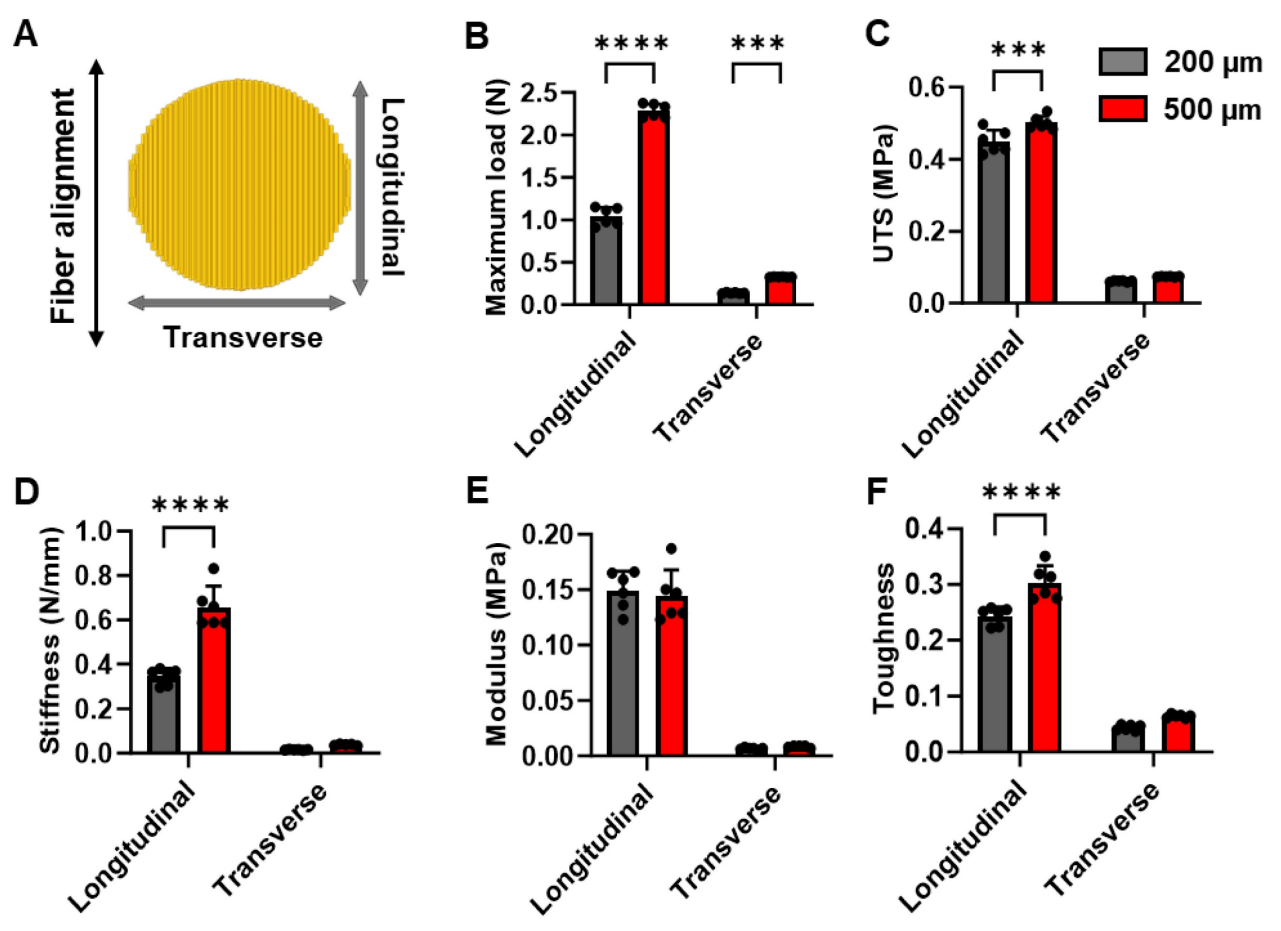
** Assessment of the mechanical properties of PCL-gelatin patches of different thickness. (A)** Schematic representation of fiber alignment, and direction of transverse and longitudinal force applied to the patches for mechanical testing. Quantitative assessment of **(B)** maximum load, **(C)** ultimate tensile strength (UTS), **(D)** stiffness, **(E)** modulus, and **(F)** toughness of the 200 µm and 500 µm thick patches along the longitudinal and transverse directions. N=6, ***: p<0.01, ****: p<0.001.

To determine if the thickness of the scaffolds influenced their mechanical properties, we assessed the strength of the 200 µm and 500 µm thick scaffolds along the longitudinal (parallel to fiber alignment) as well as transverse (perpendicular to fiber alignment) directions (**Fig. 2A**). We observed a significant, more than 2-fold increase in the maximum load that can be carried by the 500 µm scaffolds as compared to 200 µm scaffolds along both the longitudinal as well as transverse directions (**Fig. 2B**). Furthermore, we observed a significant increase in the ultimate tensile strength (UTS), stiffness, and toughness of the 500 µm scaffolds when compared to the 200 µm scaffolds along the longitudinal direction (**Fig.2C, D, F**). However, no significant differences were observed in the transverse direction. On the other hand, we did not observe any significant differences in the Young's modulus between the two scaffolds along the longitudinal or transverse directions (**Fig. 2E**). Taken together, our mechanical testing experiments demonstrated that the 500 µM scaffolds had superior mechanical properties and would be able to withstand a higher load.

### 3.2. *In vitro* assessment of hiPSC-CM morphology and function on cardiac patch

**Figure 3:**
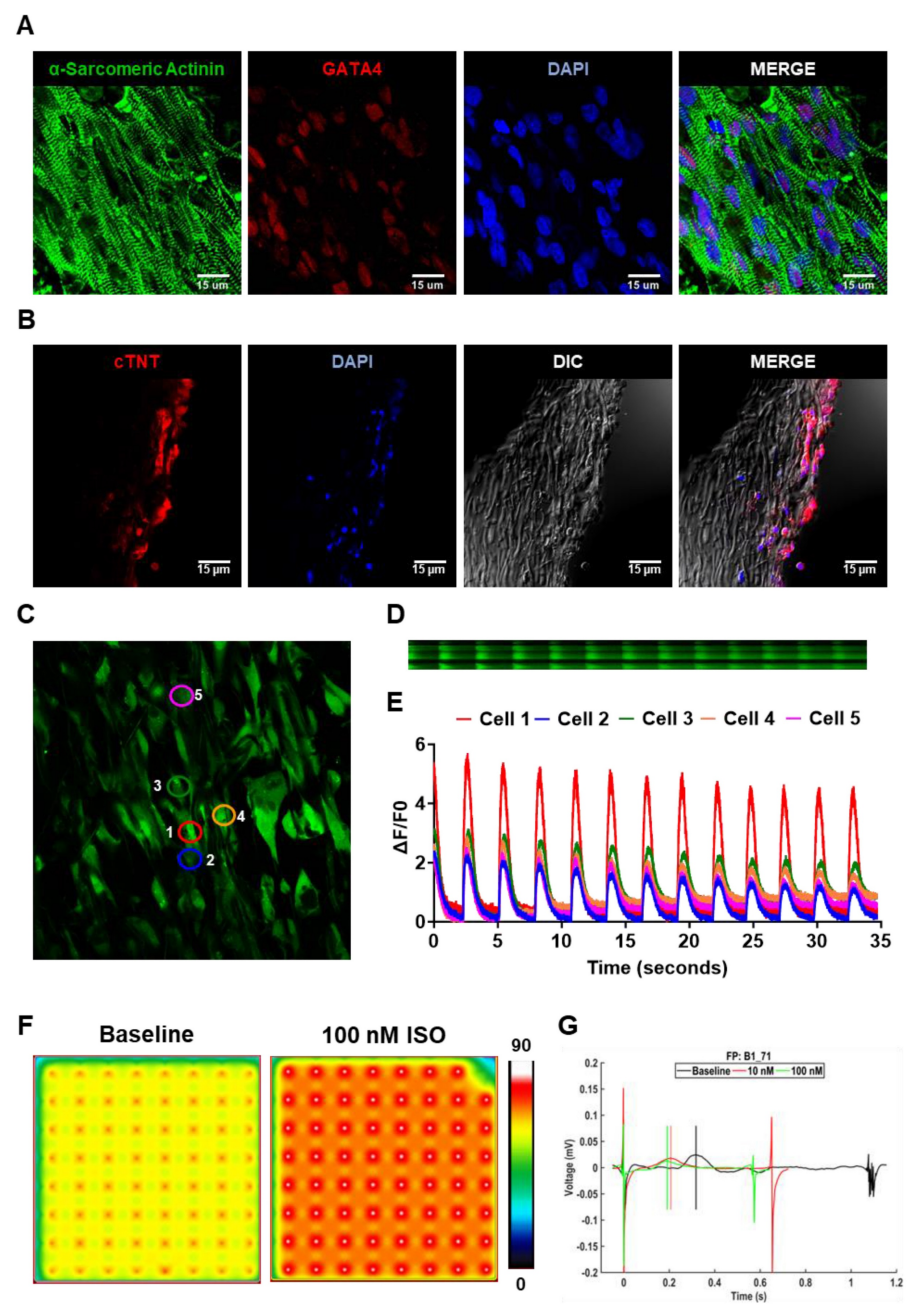
** Characterization of hiPSC-CMs cultured on aligned PCL-gelatin coaxial nanofiber patch. (A)** Confocal images showing expression of α-sarcomeric actinin and GATA4 in hiPSC-CMs cultured on aligned PCL-gelatin nanofiber patches. **(B)** Confocal images showing expression of cardiac troponin-T (cTnT) in cross-section of PCL-gelatin nanofiber patches seeded with hiPSC-CMs on both sides. Scale: 15 µm. **(C)** Representative field showing calcium transients in hiPSC-CMs cultured on aligned nanofiber patches**. (D)** Representative images showing line scan images of calcium transients in five cells and their corresponding quantification **(E). (F)** Representative heat map images showing beat rate (beats per minute) of hiPSC-CMs cultured on aligned nanofiber patches at baseline and following treatment with 100 nM isoproterenol (ISO). **(G)** Representative image showing field potentials of hiPSC-CMs cultured on aligned nanofiber patches at baseline and following treatment with 10 nM and 100 nM ISO.

The hiPSC-CMs were cultured on aligned coaxial fiber patches, and their morphology was assessed at five days. The hiPSC-CMs appeared elongated in morphology (**Fig. 3A**) and were arranged in parallel to the fibers. Furthermore, we observed highly aligned sarcomeres in these hiPSC-CMs as observed by immunostaining for α-sarcomeric actinin (**Fig. 3A**). We also observed the expression of GATA4 in all the hiPSC-CMs (**Fig. 3A**). However, while we observed a uniform distribution of the hiPSC-CMs on the surface of the patches, cross-section images of the cardiac patch showed the absence of migration of the hiPSC-CMs into the patch (**Fig. 3B**). We observed the presence of cTNT-expressing hiPSC-CMs only along the surface of the patch on both sides (**Fig. 3B**).

Furthermore, we assessed the functionality of the hiPSC-CMs cultured on these aligned patches via fluorescence-based monitoring of calcium transients in these cells. Confocal microscopy showed synchronous calcium cycling in the hiPSC-CMs cultured on the aligned fiber patches (**Fig. 3C**). Furthermore, quantification of line scan images of neighboring hiPSC-CMs on the patch (**Fig. 3D**) showed synchronous increase and decrease of intracellular calcium levels in these cells (**Fig. 3E**). Additionally, we also assessed the function of hiPSC-CMs cultured on the aligned fiber patches using the MEA system (**Fig. 3F-G**). MEA analysis showed a significant increase in beat rate (beats per min) in the hiPSC-CMs cultured on the fiber patches following 10 nM and 100 nM ISO-treatment as compared to the baseline (**Fig. 3F-G**). Taken together, our observations showed the presence of a functional syncytium in the hiPSC-CMs cultured on the aligned fiber scaffolds.

### 3.3. Electrocardiographic and biochemical assessment of cardiac injury and arrhythmias

**Figure 4:**
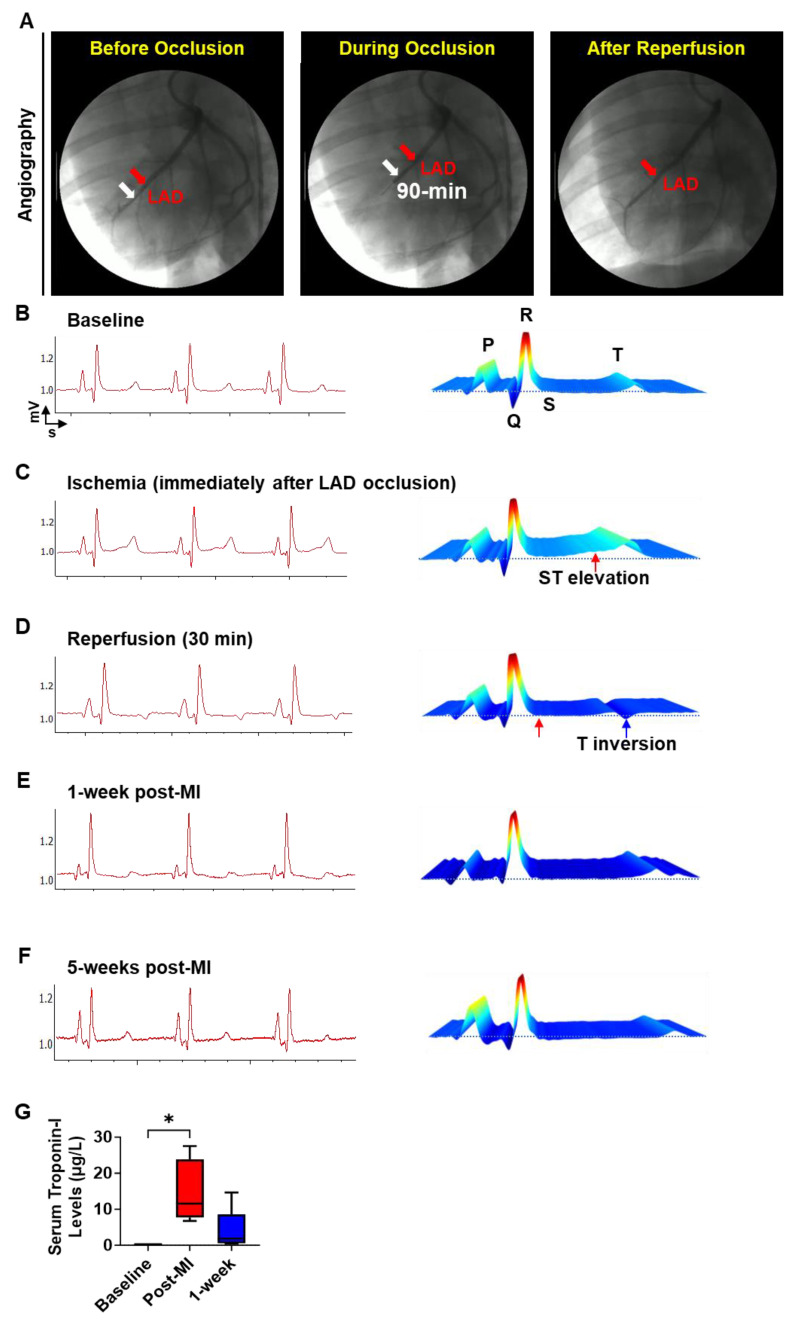
** Induction of MI in Yorkshire pigs. (A)** Fluoroscopic images showing LAD occlusion for induction of MI. Red arrow: maneuvering wire; White arrow: inflated balloon. **(B-F)** Representative echocardiogram **(B-F)** showing cardiac function and the corresponding waterfall plots at Baseline **(B)**, Ischemia **(C),** immediately after MI **(D)**, 1-week post-MI **(E),** and 5-weeks post-MI **(F)**. **(G)** Serum troponin-I levels at baseline, 90 min after LAD occlusion, and 1-week after MI induction. N = 5, p < 0.05.

To establish a translationally relevant preclinical porcine MI model, an appropriately sized coronary angioplasty balloon catheter (indicated by white arrows) was positioned in the left anterior descending (LAD) artery (indicated by red arrows) just distal to the first diagonal branch (**Fig. 4A**). The LAD was occluded for 90 min by inflating the balloon to induce ischemic injury. For reperfusion, the balloon was deflated and retracted from the LAD as shown in **Fig. 4A**. Induction of IR injury and occurrence of cardiac arrhythmias were evaluated via ECG (**Fig. 4B-F**). Baseline ECG traces and water fall plot showed a normal sinus rhythm characterized by a P wave, QRS complex, ST segment and T wave indicating normal electrical conduction in the cardiac muscle (**Fig. 4B**). After LAD occlusion via balloon inflation, we observed an ST-elevation indicating the induction of ischemia in these hearts (**Fig. 4C**). Following the retraction of the deflated balloon, we observed a T wave inversion in the ECG, showing the successful induction of MI (**Fig. 4D**). Moreover, we did not observe significant changes in the ECG or any incidents of arrhythmia at 1-week post-MI, when the patch was transplanted (**Fig. 4E**), or at 5-weeks post-MI (**Fig. 4F**). Corresponding to the changes in ECG, we observed a significant increase in serum troponin-I levels **(Fig. 4G)** immediately after IR injury indicating cardiomyocyte death. While the troponin-I levels in circulation remained high for 1-week after IR injury, the levels were lower than what we observed immediately after injury. Taken together, our data demonstrates that the induction of IR resulted in a significant cardiac injury and that the cardiac patch transplantation did not induce any arrhythmia in the pigs.

### 3.4. Assessment of cardiac function

**Figure 5:**
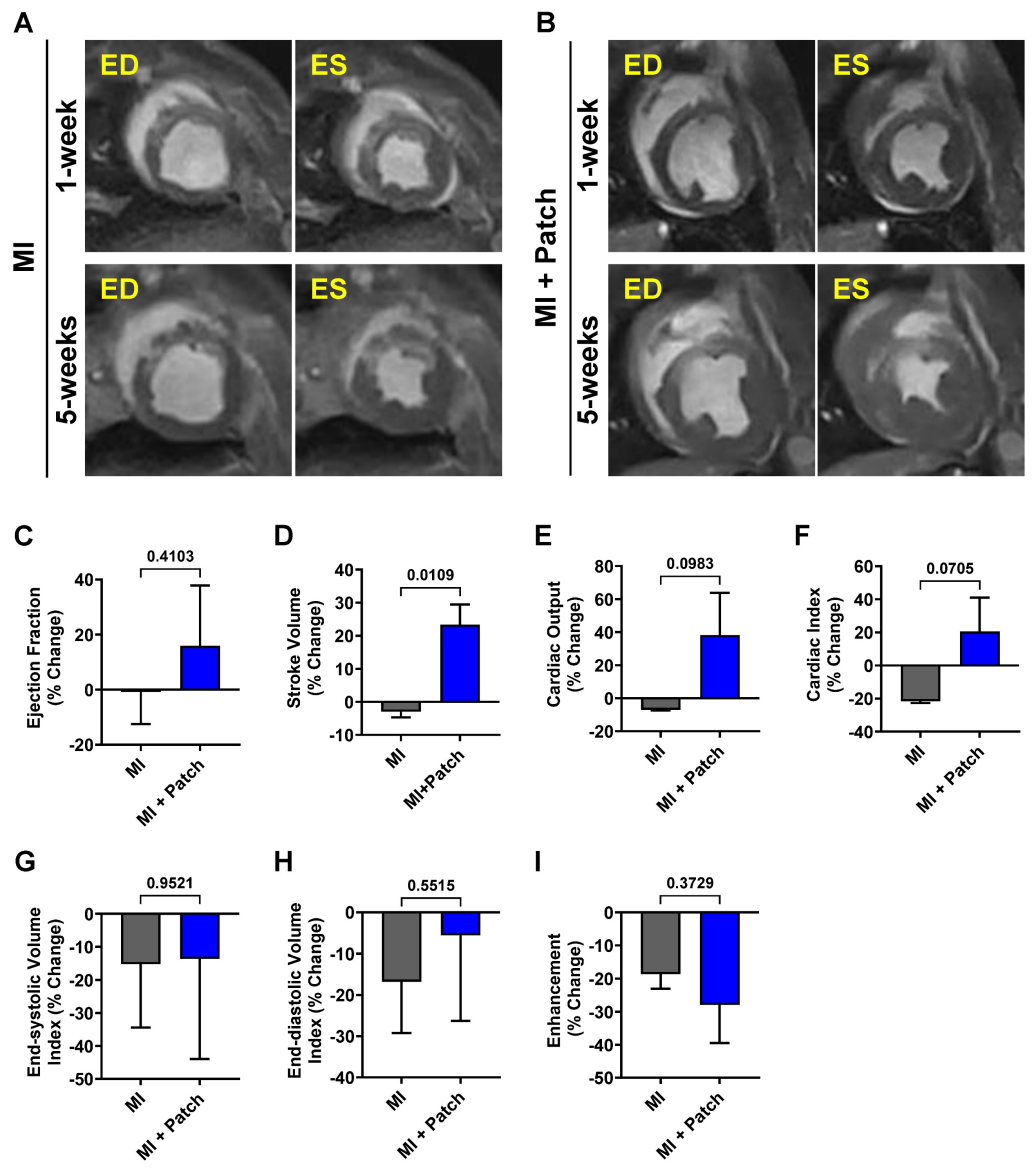
** Assessment of changes in cardiac function post-patch transplantation in pig MI model.** Representative short-axis cine images of the mid-apical slice of **(A)** Control (MI-only) and **(B)** patch-transplanted pig (MI+ Patch) at 1-week and 5-weeks post-MI. ED: End-diastolic; ES: end-systolic. Quantitative assessment of changes in cardiac function in Control (MI-only) and patch-transplanted (MI +Patch) pigs showing percentage change in **(C)** Ejection Fraction, **(D)** Stroke Volume, **(E)** Cardiac Output, **(F)** Cardiac Index, **(G)** End-diastolic Volume Index, **(H)** End-systolic Volume Index, **(I)** Heart Rate, and **(J)** Enhancement Percentage at 5-weeks (post-MI) compared to 1-weeks (post-MI). N=2 in MI-only, N=3 in MI + Patch group.

Changes in cardiac function in control (MI-only) and patch-transplanted (MI + Patch) pigs were assessed using MRI (**Fig. 5**, **Supplemental Table 2**). We observed an 18.0% increase in ejection fraction (EF) in the MI + Patch group, while the MI-only group showed a slight decline of 1.2% at 5-weeks post-MI compared to 1-week post-MI. Similarly, compared to the MI-only group, the MI+ patch group showed an increase in cardiac output and cardiac index at 5-weeks post-MI. We did not see significant differences between the groups with respect to end-systolic volume index and end-diastolic volume index.

### 3.5. Assessment of infarction using magnetic resonance imaging with late gadolinium enhancement

**Figure 6:**
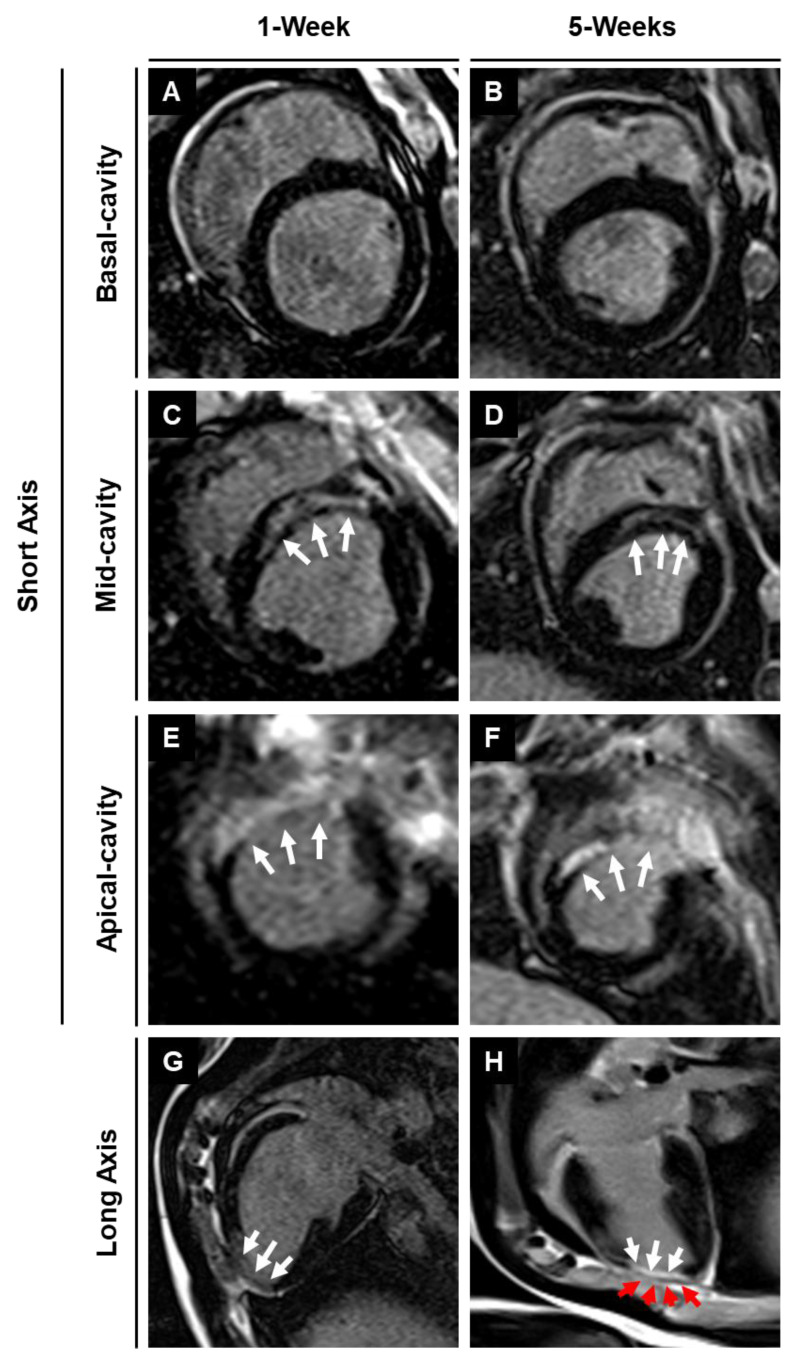
** MRI-LGE images showing the site of patch transplantation on the pig heart.** Representative LGE images along the** (A-F)** short and** (G-H)** long axis at 1- and 5-weeks post-MI in MI + Patch group. White arrows are pointing towards the hyper enhanced infarcted region involving the left ventricular apical wall and interventricular septum. Red arrows are pointing towards the transplanted epicardial patch over the infarcted region (dark area on LGE imaging) **(H)**.

Late gadolinium enhancement imaging was used to identify the location and extent of cardiac fibrosis in the infarcted area and subsequent scar tissue remodeling over 5-weeks post-MI. LGE imaging along the long axis showed the location of the transplanted patch appearing as dark epicardial hypointense area, and the area of infarct which appeared as an area of hyper-enhanced myocardium (**Fig. 6**). LGE imaging along the short axis demonstrated the localization of the infarct region at the anteroseptal wall in the mid and apical regions, revealing an epicardial/mid-wall pattern of LGE consistent with non-transmural infarction **(Fig. 6)**. Furthermore, quantitative assessment showed a 28.8% reduction in infarct size by 5-weeks post-MI in pigs treated with the cardiac patch. In contrast, pigs that did not receive the cardiac patch (control group) exhibited a slight decrease in infarct size by 18.6%. While the reduction in infarct size in the pigs that received the cardiac patch transplant was higher than the control animals, the difference was not statistically significant between the two groups. We observed a slightly higher decrease in enhancement percentage in the MI + patch group, indicating a smaller region of fibrosis in these animals compared to the MI-only group.

### 3.6. Assessment of fibrosis and engraftment of transplanted hiPSC-CMs

**Figure 7:**
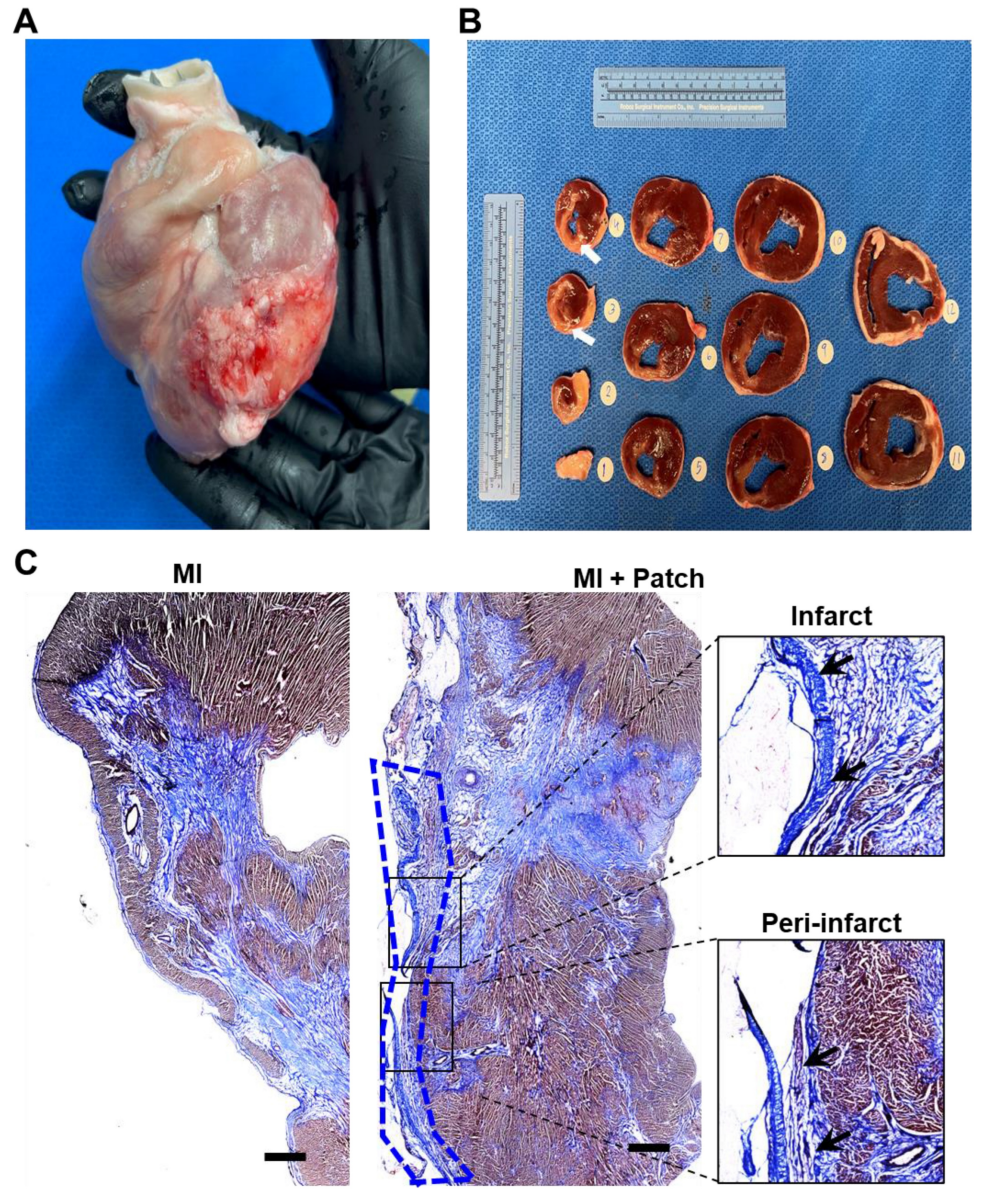
** Assessment of fibrosis at 4-weeks after patch transplantation. (A)** Whole pig heart showing site of patch engraftment at 4-weeks. **(B)** Heart sections showing infarct region and transplanted patch integration. **(D)** Masson-trichrome staining showing fibrosis in control and patch-transplanted pigs. Scale: 1mm.

**Figure 8:**
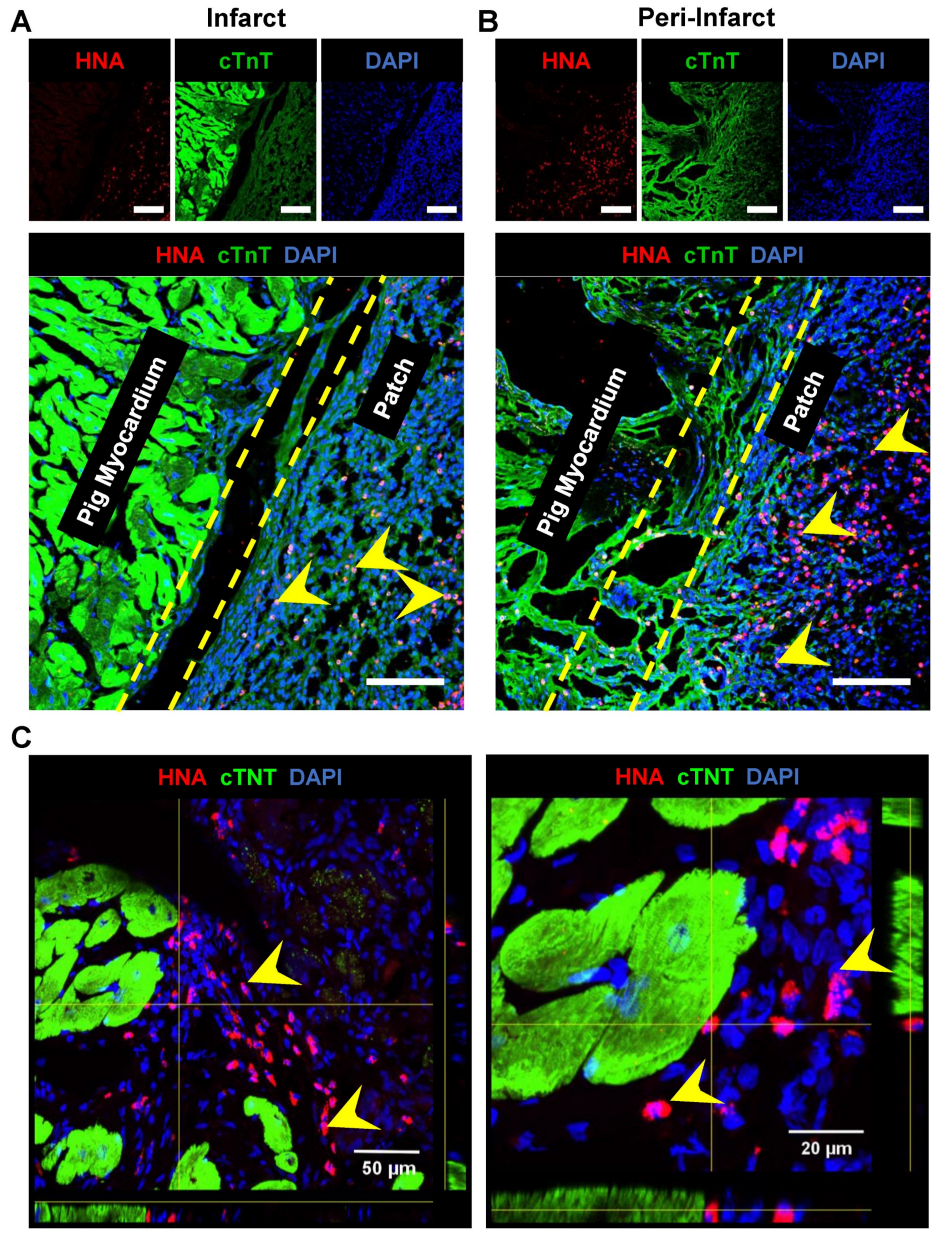
** Survival and engraftment of transplanted cardiac patch in porcine ischemic hearts**. Immunohistochemistry analysis of porcine heart section at four-weeks post cardiac patch transplantation showed survival of hiPSC-CMs (Human nuclear antigen, Red, indicated by yellow arrows) at the **(A)** infarct and **(B)** peri-infarct regions. Scale: 100 µm. **(C)** Z-stack images of periinfarct regions showing presence of human nuclei near the pig cardiomyocytes.

The pigs were euthanized, and the hearts were collected at 4-week post-patch transplantation (**Fig. 7A**). We did not observe degradation of the transplanted patch for 4 weeks. Additionally, we observed significant infarction in the septum of the porcine hearts post LAD occlusion (**Fig. 7B**). The infarct was also observed along the left ventricular wall (**Fig. 7B**). Assessment of the fibrosis in the left ventricle near the patch-transplant region confirmed absence or reduction in fibrosis near the patch (**Fig. 7C**). Furthermore, immunohistochemistry confirmed the presence of transplanted hiPSC-CMs at 4-weeks post-transplantation, in both the peri-infarct and infarct regions, as evidenced by the positive staining of human nuclei antigen (HNA) in these cells (**Fig. 8**). Additionally, we observed the integration of the transplanted cells in the cardiac patch with the host myocardium in the peri-infarct region (**Fig. 8B**) Moreover, Z-stack images of the peri-infarct regions showed the presence of human cells in proximity to the pig cardiomyocytes indicating a higher engraftment of the transplanted cells with the host tissue in this region (**Fig. 8C**).

## 4. Discussion

In the current study, we fabricated a large, functional bioengineered cardiac patch using a combination of hiPSC-CMs and electrospun aligned coaxial PCL-gelatin patches. To evaluate the ability of the cardiac patch to enhance the retention and survival of the transplanted hiPSC-CMs in the ischemic myocardium, we developed a clinically relevant preclinical porcine model of MI. Following epicardial transplantation of the cardiac patch at one-week post-MI, we did not observe any adverse cardiac events in the pigs up to five-week post-MI. Although not significant, preliminary trends indicated an overall improved global cardiac function in pigs at four-weeks post-patch transplantation, as observed by the enhanced cardiac output and EF. Additionally, the absence of teratomas at the end of the study established the safety of the cardiac patch. Furthermore, we identified the presence of engrafted hiPSC-CMs in the infarct and peri-infarct regions of the left ventricle at four-weeks demonstrating the potential use of the cardiac patch for retention and survival of transplanted hiPSC-CMs in a preclinical large animal model.

Cardiac regenerative therapies involving hiPSCs and hiPSC-CMs have shown promising potential as an alternative to heart transplant for patients with MI and end-stage heart failure^4^, ^28^, ^29^. However, a critical barrier in the successful translation of hiPSC-CMs to the clinic has been their poor engraftment and the long-term survival of the transplanted cells post-transplantation into the ischemic myocardium^30^-^34^. In our study, we addressed this limitation with the use of bioengineered coaxial PCL-gelatin patches seeded with hiPSC-CMs. Our data showed the survival and retention of the transplanted hiPSC-CMs within the patch for up to 4 weeks after transplantation (**Fig. 8**). Given the significantly lower number of hiPSC-CMs (~22 million) transplanted in our study as compared to previous studies^35^, ^36^, the presence of human nuclei within the transplanted patch up to 4-weeks post-transplantation showed ability of the patch to retain the hiPSC-CMs at the site of transplantation. While this could be attributed to the use of a well-optimized immunosuppressive regime, another plausible reason for this could be the unique structure and design of the patches used in our study.

Bioengineered scaffolds and hydrogels have been previously used to enhance the survival and engraftment of transplanted cells^37^-^40^. However, in addition to aiding cell retention, these scaffolds have been shown to provide additional mechanical support to the fibrotic ischemic myocardium^41^. Therefore, the selection of appropriate biomaterials which can match the mechanical properties of the native myocardium while simultaneously having a high biocompatibility becomes critical to fabricate scaffolds for cardiac applications^11^, ^37^, ^40^, ^41^. In our study, we fabricated a coaxial PCL-gelatin patch via electrospinning to obtain parallelly arranged fibers within the patch. Moreover, each fiber had a coaxial morphology with PCL and gelatin arranged in a core-shell structure (**Fig. 1**). This unique coaxial morphology allowed us to harness the biomimetic properties of gelatin in the shell, as well as the mechanical strength of PCL in the core^11^, ^25^. Our data is consistent with previous studies which showed that modification of synthetic polymer-based nanofiber surface enhances its biocompatibility and cell adhesion properties^42^-^44^.

Moreover, the use of electrospinning for the fabrication of these patches enabled us to tune the mechanical properties of the scaffold without modifying their chemical or cellular composition (**Fig. 2**). Previous studies have demonstrated the need for larger, thicker and stiffer patches to withstand the higher mechanical load of the pig myocardium as compared to mice and rats^32^, ^45^, ^46^. In addition to the differences in the size of the hearts in pigs and mice, the higher mechanical load in the porcine hearts was attributed to the significantly thicker ventricular wall (10-12mm) in pigs as compared to mice (1-2 mm). Previous studies achieved enhance patch stiffness by altering the material composition, patch dimensions, and cellular composition^32^, ^45^, ^46^. Contrarily, in our study, we demonstrated that the stiffness of the electrospun PCL-gel patches could be significantly increased by changing their overall thickness from 200 µm to 500 µm. Moreover, the increased thickness did not significantly alter the hiPSC-CM morphology or function when cultured on thicker patches (**Fig. 3**) as compared to thinner patches, as published earlier^11^.

The clinical application of hiPSC-CMs for cardiac regenerative medicine has also been impeded by two major safety concerns, the plausible tumorigenic and arrhythmogenic risk associated with the presence of remnant undifferentiated hiPSCs or progenitor cells, and poor integration with the host myocardium^38^, ^47^. Firstly, in our study we did not observe any tumorigenic growth at the site of the patch transplantation at 4-weeks post transplantation (**Fig. 7**). Since undifferentiated hiPSCs can form teratomas within 4-weeks, our observation reaffirms the safety in using terminally differentiated hiPSC-CMs-seeded onto the cardiac patch^48^-^53^. However, it is imperative to assess the purity of hiPSC-CMs to ensure the absence of contaminating hiPSCs or progenitors prior to in vivo transplantation studies, especially when using higher cell doses^38^. Secondly, ECG analysis showed the absence of arrhythmias in the pigs up to 4 weeks after patch transplantation (**Fig. 4**). While continuous assessment of cardiac function may be required to firmly establish the absence of arrhythmias, our observation shows that the cardiac patch does not disrupt the electrical conduction in the pig hearts. Future studies using telemetric device implantation and optical mapping studies will be carried out to determine the functional integration of the epicardially transplanted hiPSC-CMs with the host myocardium.

Use of translationally relevant preclinical animal models is essential to the successful translation of therapies from bench to bedside^54^-^56^. The similarity in the size and anatomy of pig hearts with that of human hearts makes them an ideal choice for preclinical studies^54^. However, most of these studies are designed to deliver cell-based therapies immediately after induction of MI, which does not accurately mimic the disease pathology and heterogeneity of MI patients in the clinic^56^. In our study, we developed a translationally relevant porcine model wherein we transplanted the hiPSC-CM-seeded cardiac patch at one-week post-MI to allow sufficient time for the initiation of early cardiac remodeling and initiation of fibrosis, as evidenced by the hyper enhanced region in the LGE images at one-week (**Fig. 6**). Moreover, the interval between MI induction and cell transplantation increased the heterogeneity in the infarct size among the pigs used in the study (Supplementary Table 2). Recent investigations in mini pigs and non-human primates have demonstrated improved clinical translatability when cell-based therapies are administered after the onset of early or late cardiac remodeling^38^, ^57^, ^58^. Therefore, the design and outcomes of our study may provide higher potential for future efficacy studies and translation to clinical trials.

### 4.1. Limitations and future directions

While the primary goal of our study was to evaluate the safety and engraftment of the transplanted hiPSC-CMs in a porcine MI model, MRI-based assessment of the cardiac function in pigs at the end of the study showed a small improvement in function in animals with patch transplantation. However, despite these promising preliminary trends, the functional outcomes from our study were not statistically significant. While this limited beneficial effect of the cardiac patch could be attributed to the small sample cohort and low dose of transplanted cells (22 million, 0.7-1 × 10^6^/kg bodyweight) as compared to other studies using significantly higher doses of cells (8-20× 10^6^/kg bodyweight)^38^, another key factor could be the spatial mismatch between the site of infarct and site of patch transplantation (**Fig. 6 and 7**). Both MRI and histology data showed the formation of an anterior-septal infarct in the pigs following LAD occlusion. On the other hand, the cardiac patch was transplanted on the epicardial surface near the lateral wall of the pig hearts, which is distinct from the region of necrotic tissue. Therefore, it is plausible that this spatial mismatch may have limited the regenerative potential of the transplanted hiPSC-CM-seeded cardiac patch and the modest improvement in cardiac function may be due to paracrine signaling from the surviving hiPSC-CMs rather than direct remuscularization, as reported by previous studies^38^, ^58^, ^59^. To overcome these limitations, MI induction in pigs would have to be optimized to establish a transmural infarct involving full wall thickness, which is more prevalent in the clinical settings and is associated with a higher risk of mortality^60^. A few studies have reported the development of a transmural infarct in pigs following occlusion of the left circumflex (LCX) artery^61^-^63^. Therefore, follow-up studies will be focused towards optimizing the LCX occlusion model to establish a transmural infarct in pigs for testing epicardial patch transplantation. Lastly, whether the beneficial outcomes of the cardiac patch were due to the paracrine signaling by hiPSC-CMs or via structural support imparted by the patch is yet to be determined. To delineate the specific contribution made by the patch material and the hiPSC-CMs, respectively, and to establish the efficacy of the cardiac patch, future studies will evaluate the effect of “only patch” transplantation, assess the dose-response of transplanted hiPSC-CMs, and determine the long-term functional outcomes using a larger cohort of animals.

## 5. Conclusion

In conclusion, our study provides compelling evidence supporting the safety and successful engraftment of transplanted PCL-gelatin cardiac patch seeded with hiPSC-CMs in a preclinical porcine MI model. These patches demonstrated promising integration with the host tissue, with viable hiPSC-CMs surviving in the ischemic myocardium for up to 4-weeks post-transplantation. The modest improvement in cardiac function observed in this study suggests a preliminary, yet promising, beneficial effect of the transplanted patch. However, future studies involving a larger cohort of animals, longer study duration, and higher dose of hiPSC-CMs will be required to establish the efficacy of the cardiac patch. Lastly, in addition to its application for assessing the global and regional changes in cardiac function, the use of MRI with LGE imaging could serve as a valuable tool for accurate non-invasive characterization of the infarct location and extent, prior to patch transplantation.

## Supplementary Material

Supplementary figures and tables.

## Abbreviations

ATR-FTIR: Attenuated total reflectance-Fourier transform infrared spectroscopy
CABG: Coronary artery bypass grafting
ECG: Electrocardiogram
EDC: 1-ethyl-3-(3Dimethylamino propyl) carbodiimide hydrochloride
EHT: Engineered heart tissues
hiPSC-CMs: Human induced pluripotent stem cell-derived cardiomyocytes
IR: Ischemia-reperfusion
ISO: Isoproterenol
LAD: Left anterior descending artery
LCX: Left circumflex artery
LGE: Late gadolinium enhancement
LV: Left ventricle
MI: Myocardial infarction
MRI: Magnetic resonance imaging
PCI: Percutaneous coronary intervention
PCL: Polycaprolactone
PDMS: Polydimethylsiloxane
PLA: Poly (L-lactic acid)
PLGA: Poly (lactic-co-glycolic acid)
SEM: Scanning electron microscopy
TEM: Transmission electron microscopy
