# Supplementary material for: Safety and Engraftment of Aligned Cardiac Patches Loaded with hiPSC-CMs in a Large Animal Model of Myocardial Infarction
Source: Theranostics. 2026 Jan 14;16(8):3978–95. doi: 10.7150/thno.121105 (PMC12905669; doi:10.7150/thno.121105)
Supplement: Supplementary file 1 — Supplementary figures and tables. [file thnov16p3978s1.pdf]

Supplementary Information

Safety and Engraftment of Aligned Cardiac Patches Loaded with hiPSC-CMs in a Large Animal Model of Myocardial Infarction

Divya Sridharan[1,2], Salman Pervaiz[2,3], Nikita C. Nair[2], Muhamad M. Mergaye[1,2], Helena Islam[1,2], Britani N Blackstone[4], Syed A. Ashraf[1, 2,] Syed B. Alvi[1,2], Matthew Joseph[2], Juliet Varghese[4], Yuchi Han[2,3], Orlando P. Simonetti[2,3,4,5], Heather M. Powell[4], Konstantinos Dean Boudoulas[3], Robert L. Hamlin[6] and Mahmood Khan[1,2*]

Supplementary Fig. 1

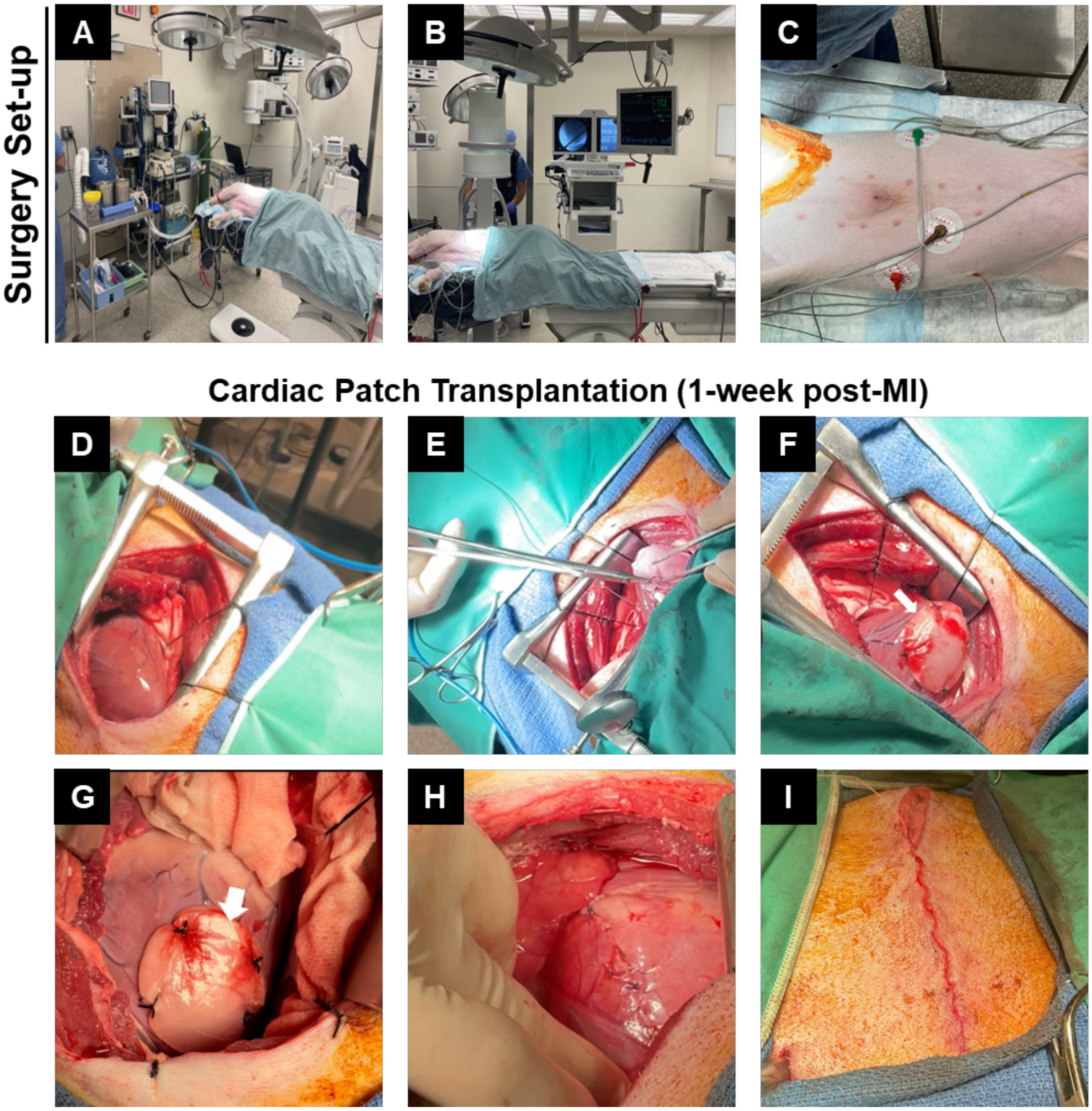

Supplementary Fig. 1. Surgery setup. (A-C) Surgery set-up for induction of MI. **(D)** Image showing opening of porcine chest to identify region of infarction. **(E-F)** Images showing suturing of cardiac patch onto the epicardial surface of the infarcted heart. **(G)** Cardiac patch sutured on epicardial surface of the heart. **(H)** Suturing of pericardium onto the heart. **(I)** Chest closure.

Supplementary Table 1. Age and body weights of the pigs used in the study.

Pig ID	Age at the start of Exp (days)	Weight before MI (Baseline)	Weight at 5-weeks
162	~ 3 months old	32.0 kg	50.4 kg
5142	~ 3 months old	28.4 kg	40.0 kg
112	~ 3 months old	32.6 kg	42.6 kg
184	~ 3 months old	36.6 kg	43.6 kg
5143	~ 3 months old	27.4 kg	38.0 kg

Supplementary Table 2. MRI assessment of cardiac function and LGE parameters

Parameter	MI-only (n=2)		MI + Cardiac Patch (n=3)	
	1-week	5-weeks	1-week	5-weeks
Ejection fraction (%)	42.0±2.8	41.5±2.1	33.3±5.5	39.3±12.7
Stroke Volume (ml)	48.7±12.5	47.2±11.2	36.3±8.8	44.5±9.0
Stroke Volume Index (ml/m^2)	45.3±7.6	40.0±4.9	33.6±6.2	36.0±5.9
End-diastolic Volume (ml)	116.6±37.4	112.5±21.9	108.7±9.3	116.6±19.6
End-diastolic Volume Index (ml/m^2)	108.1±25.4	88.3±7.7	101.4±6.9	96.1±24.1
End-systolic volume (ml)	67.8±24.8	65.4±10.8	72.5±0.8	72.2±27.1
End-systolic Volume Index (ml/m^2)	62.7±17.7	51.5±2.9	67.9±7.4	60.2±27.4
Cardiac Output (L/min)	4.3±0.3	4.0±0.3	3.4±0.7	4.6±0.4
Cardiac Index (L/min/m^2)	4.0±0.1	3.2±0.1	3.2±0.5	3.8±0.1
Enhancement Mass (g)	8.8±2.1	7.6±1.0	7.6±3.0	7.5±2.7
LV Mass (g)	60.1±5.2	64.5±1.4	61.3±17.7	86.4±0.6
Enhancement percentage (%)	14.5±2.3	8.9±3.7	12.5±5.2	11.8±1.2
